# Central Nervous System Targets: Supraspinal Mechanisms of Analgesia

**DOI:** 10.1007/s13311-020-00887-6

**Published:** 2020-07-22

**Authors:** K. Bannister, A. H. Dickenson

**Affiliations:** 1 Department of Pharmacology and Therapeutics, Institute of Psychiatry, Psychology and Neuroscience, Wolfson CARD, Guy’s Campus, King’s College London, London, SE1 1UL UK; 2 Department of Neuroscience, Physiology and Pharmacology, University College London, London, WC1E 6BT UK

**Keywords:** Supraspinal, Analgesic, Anticonvulsant, Opioidergic, Antidepressant

## Abstract

While the acute sensation of pain is protective, signaling the presence of actual or potential bodily harm, its persistence is unpleasant. When pain becomes chronic, it has limited evolutionarily advantage. Despite the differing nature of acute and chronic pain, a common theme is that sufferers seek pain relief. The possibility to medicate pain types as varied as a toothache or postsurgical pain reflects the diverse range of mechanism(s) by which pain-relieving “analgesic” therapies may reduce, eliminate, or prevent pain. Systemic application of an analgesic able to cross the blood–brain barrier can result in pain modulation via interaction with targets at different sites in the central nervous system. A so-called supraspinal mechanism of action indicates manipulation of a brain-defined circuitry. Pre-clinical studies demonstrate that, according to the brain circuitry targeted, varying therapeutic pain-relieving effects may be observed that relate to an impact on, for example, sensory and/or affective qualities of pain. In many cases, this translates to the clinic. Regardless of the brain circuitry manipulated, modulation of brain processing often directly impacts multiple aspects of nociceptive transmission, including spinal neuronal signaling. Consideration of supraspinal mechanisms of analgesia and ensuing pain relief must take into account nonbrain-mediated effects; therefore, in this review, the supraspinally mediated analgesic actions of opioidergic, anti-convulsant, and anti-depressant drugs are discussed. The persistence of poor treatment outcomes and/or side effect profiles of currently used analgesics highlight the need for the development of novel therapeutics or more precise use of available agents. Fully uncovering the complex biology of nociception, as well as currently used analgesic mechanism(s) and site(s) of action, will expedite this process.

## Introduction

Nociception refers to the way in which peripheral and central nervous system circuits process information following activation of peripherally located nociceptors. Thereafter, the perception of a noxious stimulus is a centrally driven event, and central nervous system mechanisms can significantly alter our experience of pain. There exist a broad range of analgesic therapies that may act to reduce, eliminate, or prevent the short or long-lasting pain associated with varied pain types. This is possible because of distinct (and sometimes multiple) mechanisms of action. In common for all analgesic therapies is exploitation of our naturally occurring biological make-up and its plasticity in disease. A deeper understanding of the biology of nociception, and dissection of the ways in which the analgesics mediate their favorable response, has aided therapeutic development. In this review, the supraspinal analgesia–mediating mechanisms of action of 3 major drug classes are considered with reference, where appropriate, to the impact of supraspinal action on peripheral and spinal processes.

## Opioidergic Analgesia

Naturally occurring opioid peptides elicit inhibitory effects that counteract the activity in excitatory pain pathways that manifests following, for example, activation of peripheral nociceptors. Highly potent synthetic opioids mimic the receptor-driven activity of the naturally occurring opioids, and the mechanism of action encompasses activity in peripheral, spinal, and supraspinal target sites. Here, the supraspinal receptor–mediated mechanisms of action of endogenous and synthetic opioids are discussed.

### Receptor-Mediated Mechanisms

The endomorphins, enkephalins, and dynorphins are widely distributed throughout the central nervous system (CNS), acting preferentially on μ, δ, and κ opioid receptors (MOR, DOR, and KOR) respectively. Naturally occurring opioids may also be found in plants (e.g., morphine). Activation of inhibitory G-protein-coupled MOR, DOR, or KOR leads to suppression of nociceptive-related signaling in central neuronal pathways [1]. On a cellular level, voltage-gated calcium channels close and potassium efflux occurs, leading to hyperpolarization. The result is reduced (1) neuronal cell excitability and thus (2) nociceptive transmission. Synthetic opioids are used clinically and exert their effect via the same receptor family. Examples of clinically relevant opioids include morphine, diamorphine, pethidine, and fentanyl. While all listed are selective for the MOR, low-affinity binding to the DOR and KOR may occur also. Compounds including norbinaltorphimine (nor-BNI) are used widely in scientific research due to preferential binding to the KOR [2].

### Supraspinal Sites of Action: Brain Stem and Midbrain

Key sites of opioid analgesia include brainstem and midbrain loci due to high receptor concentrations in the nuclei of the tractus solitarius and the periaqueductal grey (PAG) [3]. Elegant microinjection studies demonstrated that such brainstem and midbrain sites could mediate opioid-induced inhibitions when local administration of morphine elicited analgesia against applied stimuli [4–6]. Lower doses of morphine are proposed to preferentially modulate signaling in the brain circuitry since moderate doses of morphine are shown to have reduced efficacy in the presence of MOR antagonist naloxone compared to higher doses (where presumably the spinal site of action predominates) [7]. Pioneering studies implicated the rostroventral medial medulla (RVM) as a key mediator of supraspinal opioid-induced analgesia. In this brainstem region, two physiologically definable and opioid-relevant neuronal types were identified. “Off” cells were activated by morphine, inhibiting nociceptive transmission, while “On” cell activity was depressed, indicating a pronociceptive role. In the same study, systemic administration of MOR antagonist naloxone was shown to reduce the analgesic effect of morphine, confirming an MOR-mediated response [8]. Despite this, neither direct nor indirect activation of MOR-positive neurons in the RVM is required for analgesia [9]. Opioid-mediated analgesic effects in the PAG, locus coeruleus, and RVM impact the final throughput of nociceptive information to the spinal cord dorsal horn. Opioidergic and GABAergic RVM-derived descending inputs exist [10], and recently, RVM GABAergic neurons were implicated in the facilitation of mechanical pain via inhibition of spinal enkephalinergic/GABAergic interneurons [11]. Supraspinal modulation of brainstem circuits may influence descending complex inhibitory circuits in terms of a favorable effect on pain thresholds. These data highlight the complexity of the therapeutic potential of analgesics whose mechanism of action relies upon supraspinal modulation of these regions.

### Supraspinal Sites of Action: Higher Brain Centers

High densities of opioid receptors are found in higher “affective” brain centers [10, 12]. The anterior cingulate cortex (ACC) has a key role in pain processing and pain-related emotion [13, 14] and widely connects with brainstem and midbrain loci, including the RVM [15]. Endogenous opioid signaling in the ACC modulates pain aversiveness and, upon chronicity, the amygdala is a key player in affective pain. The latter is a subcortical region that shapes the emotional components of pain. Since opioid receptors are present in the central (CeA) nucleus of the amygdala, this area was proposed to contribute to the control of pain through opioid mechanisms. Symmetry between both sides of the CeA was investigated and neuronal activity was increased in the right CeA in chronic pain states [16]. Questions remain regarding the intricacies of medullo–spinal loops mediating anti-nociception and a direct relay from the CeA. While opioidergic manipulation of this brain region inhibits stress-induced pain [17], “analgesic” effects mediated at synaptic inputs onto the PAG from CeA projection neurons for example have not been confirmed, and so targeting them therapeutically is not a viable option.

### Supraspinal Sites of Action: a Whole Brain View

Understanding the unique influence of individual supraspinal circuits on the pain experience will enable the formulation of optimized therapeutic strategies for varied pain types. Interactions between sensory (traditionally viewed as spinal-thalamic-cortical and brainstem and midbrain loci) and affective (traditionally viewed as limbic and other higher brain loci) brain regions, and the impact of discrete opioid delivery in these regions on nociception, are under investigation. What is the relationship between the pain experience and activity in individual supraspinal circuits and how is this modulated in the presence of an opioidergic analgesic? Are sensory and affective qualities of pain differentially regulated by brain opioid receptor circuitries?

It has long been recognized that opioids could produce a reduction in the aversion produced by a pain condition, separable from sensory analgesia. When studying ongoing aversive states in rodent pain models, an ongoing issue was an inability to gauge analgesic responses through the application of various modalities of stimuli. To overcome this problem Porreca and colleagues established “conditioned place preference” (CPP), a paradigm in which pain is paired with a preferred choice of environment for rats previously paired with positively reinforcing drugs. When coupled with *in vivo* microdialysis, it was possible to assess negative reinforcement. Pain relief elicits reward mediated by an elevation of dopaminergic signaling in the nucleus accumbens (NAc), and NAc dopaminergic transmission and opioid receptor–mediated signaling in the ACC were deemed necessary and sufficient for relief of pain aversiveness [18] [2015]. In a recent behavioral study, morphine was microinjected into areas of the ACC or RVM and responses to applied stimuli were measured in control rats and those with nerve injury. Acute tail-flick responses and tactile allodynia were inhibited by RVM morphine producing both anti-hyperalgesic and analgesic effects against mechanical and thermal stimuli, as well as CPP selectively in nerve-injured rats. Thus RVM morphine acts to control nociceptive transmission (withdrawal responses to evoked stimuli were inhibited) yet also control affective pain behaviors. In contrast, ACC morphine failed to modulate tactile allodynia, mechanical and thermal hyperalgesia in neuropathic rats, while affective components of ongoing pain were controlled. The data suggest that opioid circuits within the RVM and ACC differentially modulate sensory and affective qualities of pain [19].

Complimenting the behavioral studies an *in vivo* electrophysiology study hypothesized a differential role for the RVM, ACC, and right CeA in regulation of spinal neuronal processes. The data published support modulation of evoked responses of spinal cord neurons upon discrete RVM morphine delivery that was enhanced in a rodent model of chronic pain. In chronicity, opioid modulation of evoked responses was shown to occur predominately through a lateralized output from the right CeA. Minimal modulation of dorsal horn responses was observed following ACC morphine administration regardless of injury state [20]. The situation is complex not least because the brain also relies on nonopioid mechanisms to downregulate sensory pain, but relief-related analgesia relies on endogenous opioid activity. This is highly relevant if considering clinical strategies for alleviating pain in the absence of a functional opioidergic system. Understanding interactions between sensory and affective brain regions, and the impact of morphine on these areas, is vital.

### The Impact of Ascending Sensory Pathways of Pain on Supraspinal Mechanisms

What is the relationship between the affective pain experience and activity in ascending circuits? The ventrobasal thalamus is a key relay in the ascending sensory pathways of pain and neuropathy produces ongoing and enhanced evoked responses in the ventral posterolateral thalamus (VPL) [21]. More recently, the possible association of effects of ACC morphine on ascending inhibition was investigated in naïve and nerve-injured rats. The authors demonstrate inhibition of evoked neuronal activity in the VPL in neuropathic animals upon ACC morphine microinjection, but no inhibition of elevated ongoing neuronal activity [20]. Cumulatively, the data support the idea that the ACC is able to modulate the ongoing aversive state. There is a clear differentiation in terms of supraspinal opioid circuit regulation of nociceptive processing and the regulation of sensory and affective components of pain are likely separate [18]. Spinal cord outputs to the brain, when considering thalamic/cortical and parallel limbic projections, are plastic; their anatomy and functionality changes in chronicity. Analgesics that are able to target the altered and abnormal sensory messaging that the brain receives will control sensory and affective aspects of the pain experience. However, the level of alteration and abnormal processing will vary not only according to the pain type that drives the sensory experience, but also according to the way in which the individual experiences pain through the emotional filters of time, i.e., the affective experience. Thus, to find the optimum analgesic, it is insufficient to consider the pain type alone.

### The Impact of Supraspinal Opioidergic Mechanisms on Descending Control Pathways

The descending pain modulatory system comprises multiple supraspinal neuronal networks. An individual’s emotional state and sensory experience will modulate, and directly impact, the final output of the top-down controls in terms of pain perception due to limbic and thalamic brain region connectivity. Opioidergic modulation of pain (resulting from engagement of opioid receptors in multiple brain regions) invariably results in an impact on descending control pathways. This is most clearly evident when considering the impact of RVM, ACC, and right CeA morphine on spinal nociceptive processing in neuropathic rats [20]. These findings demonstrate that certain actions of morphine at central sites are specific to the right CeA (opposite to the side of injury) and leads to augmentation of descending inhibition as well as modulation of the affective qualities of ongoing pain. There are connections from the CeA to the rACC and this latter area has been implicated in the control of endogenous analgesia in both animals and humans. Diffuse noxious inhibitory controls (DNIC) originate supraspinally and, when activated by a conditioning stimulus, project to the dorsal horn of the spinal cord to inhibit nociceptive processing. DNIC-conditioning stimuli decrease RVM On-cell activity [22] and a mechanism of action that includes activation of opioid receptors is postulated [23, 24]. Brainstem MOR involvement is highly likely [25, 26]. In chronicity, enhanced descending facilitation from the brain to the spinal cord are proposed mediated, in part, by KOR signaling from the right CeA that promotes diminished DNIC [27]. DNIC are also dysfunctional following sustained morphine treatment in healthy rats, where inactivation of the RVM reinstates DNIC [28]. These data support that endogenous opioids influence the endogenous descending inhibitory DNIC pathway. This directly leads to an implication regarding the best analgesic regimen that should be applied for patients suffering from, for example, medication overuse headache or opioid-induced hyperalgesia. Enabling chronic pain patients to harness their naturally occurring analgesia-promoting DNIC pathways in disease states, where DNIC functionality is compromised, is a promising therapeutic avenue. However, the circuitry is complex and multiple supraspinal targets, each with varying opioid receptor–mediated mechanisms of action, must be considered.

Increased behavioral pain sensitivity following opioid discontinuation coincides with altered descending pain modulation. Using fMRI, a study demonstrated functional coupling between the nucleus cuneiformis and the rostral ACC increased upon opioid suspension. Increased neuronal responses in the PAG and RVM among others, as well as changes in spinal pain–related patterns, demonstrate that such changes in descending pain pathways directly relate to worsened pain perception [29]. The situation is complex not least because, as mentioned, the brain relies on opioid and nonopioid mechanisms to downregulate pain.

## Anti-convulsant Analgesia

The serendipity of drug discovery, including repurposing drugs for the control of pain from other indications, includes the use of anti-convulsants as analgesics in the clinic [30]. Their use requires passage across the blood–brain barrier. Importantly, agents with necessary central actions in the treatment of, for example, epilepsy may act through varied supraspinal (and/or peripheral) sites in pain.

### Supraspinal Sites of Action: Pain Modulation

The anti-seizure agent carbamazepine, a sodium channel blocker, can reduce pain associated with, for example, neuropathy potentially through actions at central sites while also modulating abnormal sodium channel activity in peripheral nerves. Specifically, decreased neuronal hyperexcitability through modulation of voltage-gated channels is the basis for using such drugs in chronic pain states since both pain and epilepsy share increased neuronal activity as a basis. Oxcarbazepine is a new-generation anti-convulsant with known efficacy for chronic patients sub-grouped according to their evoked hypersensitivity and preservation of primary afferent fibers. The precise site of action, central or peripheral, has recently been scrutinized. In an *in vivo* electrophysiology study, systemic oxcarbazepine markedly reduced punctate mechanical-, dynamic brush-, and cold-evoked neuronal responses in the VPL and spinal cord dorsal horn of nerve-injured rats. Spontaneous activity in the VPL was inhibited also. Intraplantar injection of the active metabolite licarbazepine replicated the effects of systemic oxcarbazepine [31]. The data strongly support the concept that ongoing activity in primary afferent fibers drives spontaneous thalamic firing after spinal nerve injury and that oxcarbazepine produces a peripheral modality-selective inhibitory effect on sensory neuronal processing. Thus, even agents with necessary central actions in the treatment of epilepsy act through very different peripheral sites in pain.

This is not necessarily the case for all anti-convulsants. The anti-hyperalgesic action of the α-2 δ ligands, gabapentin and pregabalin, is attributable to upregulation of the α-2 δ-1 accessory subunit of voltage-gated calcium channels in sensory neurons and the dorsal horn of the spinal cord [32, 33]. The gabapentinoids have supraspinal mechanisms of action despite spinal actions in controlling afferent inputs. What is the supraspinal circuitry involved? Injury-specific interactions between pregabalin and RVM MOR cells is not a permissive factor for pregabalin analgesia when applied to visceral pain [34], and opioid receptors are not necessary for the antiallodynic action of pregabalin in the context of NeP pain [35]. At the supraspinal level, gabapentin engages descending inhibitory controls in the brainstem to indirectly regulate spinal nociceptive processing [36]. Interestingly, ACC gabapentin may induce a pain-relieving effect without concurrent blockade of mechanical allodynia [37]. Whether or not direct ACC microinjection of gabapentin is representative of what occurs following systemic gabapentin injection is a valid question. Intracerebroventricular injection of the same drug is rewarding in the presence of injury and reliant on the engagement of descending inhibitory controls [38]. In a centrally driven rodent pain model, pregabalin-mediated analgesia in the absence of a peripheral pathology reflects upregulation of a serotonergic facilitatory system, presumably projecting supraspinally to the dorsal horn of the spinal cord [39]. Further evidence implicating higher brain center involvement in gabapentinoid-mediated analgesia was provided by a study that quantified CeA evoked and spontaneous activity. Both were increased in nerve ligated versus control rats and systemic pregabalin reduced the hyperexcitability in this brain region that was associated with disease progression [16].

## Anti-depressant Analgesia

Several anti-depressants are efficacious in the management of chronic pain, exerting their pain-relieving effects via a central impact on monoaminergic neurotransmission. Numerous forward and back translational studies have revealed the benefit of targeting serotonergic and adrenergic descending modulatory neurotransmission in pain relief. This mechanism is shared with the tricyclic and selective serotonin and/or noradrenaline reuptake inhibitor (SSRIs/SNRIs/NRIs) anti-depressant drugs. The clinical relevance is clear when considering that different chronic pain states may be maintained and/or amplified by dysfunctional descending monoaminergic neurotransmission [40].

### Supraspinal Sites of Action: Pain Modulation

Descending monoaminergic pathways project from the brainstem to the spinal cord and have a complex bi-directional modulatory impact on an individual’s pain experience. The actions of noradrenaline (NA) and serotonin (5HT), released by descending control pathways in the spinal cord, are chiefly implicated in nociception or anti-nociception according to the receptor that is activated. The classic premise is that spinal nociceptive processing is inhibited upon activation of the brainstem LC, which leads to activation of inhibitory spinal α-2 adrenoceptors [41]. The story with 5HT, released in the spinal cord upon activation of the RVM, is more complex due to the myriad of receptors (excitatory and inhibitory) that it may activate [42, 43].

The tricyclic anti-depressants (TCAs), including imipramine and amitriptyline, are potent NRIs. Their potential analgesic properties were recognized as early as the 1960s but it took a further 30 years before the underlying analgesic mechanism of action was elucidated [44]. SSRI/SNRIs largely replaced the TCAs due to comparable efficacy with a more appealing side effect profile [45]. Supraspinally mediated mechanisms of anti-nociception has been postulated [46] [2008].

Conditioned pain modulation (CPM) is the human counterpart of DNIC. The monoaminergic system influences the final expression of DNIC [47] and forward and back translational studies reveal the benefit of targeting serotonergic and adrenergic descending modulatory neurotransmission in pain relief. Underlying noradrenergic mechanisms explain the relationship between dysfunctional conditioned pain modulation (CPM), the human counterpart of DNIC, and the beneficial use of tapentadol (μ-opioid receptor agonist and NRI) and duloxetine (SNRI) [48, 49]. This back-translates that NRIs reinstate functional DNIC expression in neuropathic rats [50]. The central mechanism of action of tapentadol involves the spinal cord as a key site. A shift in predominantly opioid-mediated inhibitory controls in healthy animals, to a predominant noradrenergic inhibition in nerve-injured animals, is postulated [51]. Pharmacologically, induction of analgesia by brainstem–spinal cord noradrenergic pathways reflects actions at α-2 adrenoceptors (ARs) located in the dorsal horn [42]. However, this simplistic description belies the complex mechanisms involved and the precise expression profile of spinal α-2 or α-1 ARs remains unclear. Opposing α-2 AR-mediated facilitatory signaling in the brainstem [52], and the role of the LC as a chronic pain generator [53], highlights the complexity of the role(s) of supraspinal noradrenergic nuclei in the transition from acute to chronic pain, and its maintenance. A modular functional organization of the LC has been suggested [54].

Tramadol is a weak agonist of MOR, DOR, and KOR with 20-fold preference for MOR. Tramadol also combines an NRI mechanism, possessing anti-depressant properties [55]. Early indications of a central mechanism of action came from a study that demonstrated prevention of thermal hindpaw hyperalgesia (with no altered nociception) upon intraperitoneal application of low dose tramadol [56]. A supraspinal mechanism of action is postulated since human imaging studies have demonstrated enhanced reward system activation upon tramadol consumption [57] while dependency is shown to hyperexcite the motor cortex coupled with inhibitory deficits [58].

So the anti-depressants and other agents with NRI actions act primarily on the spinal terminals of descending noradrenergic fibers to restore reduced descending inhibition through this circuitry. Thus the drugs are acting at the spinal level to reactivate controls that have been switched off in the brain. One brain area controlling these systems is the amygdala [27] and cingulate cortex as well as other areas [59].

## Conclusion

Personalized analgesic approaches, for example, in terms of opioidergic targets or descending monoaminergic manipulations, will depend not only on the chronic pain type but also on the phase of the disease in question. One size will never fit all. For example, despite morphine’s central actions, it does not adequately relieve the ongoing pain and/or evoked hypersensitivities experienced by neuropathic patients. Improved analgesic development will be aided when the underlying pathological and functional mechanisms of disease states and the analgesic therapies administered respectively are fully understood. Controlled analgesic studies based on the premise that not all patients are the same, even within a defined etiology, are required. Differing sensory phenotypes may reflect different mechanisms at play in subgroups of patients. Thus, if different supraspinal mechanisms are active, then pharmacological treatments may have disparate outcomes in subgroups.
